# Investigation of anti-asthmatic potential of dried fruits of *Vitis vinifera* L. in animal model of bronchial asthma

**DOI:** 10.1186/s13223-016-0145-x

**Published:** 2016-08-17

**Authors:** Poonam Arora, S. H. Ansari, Abul Kalam Najmi, Varisha Anjum, Sayeed Ahmad

**Affiliations:** 1 Department of Pharmacognosy and Phytochemistry, Faculty of Pharmacy, Jamia Hamdard (Hamdard University), New Delhi, 110062 India; 2 Department of Pharmacology, Faculty of Pharmacy, Jamia Hamdard (Hamdard University), New Delhi, 110062 India

**Keywords:** *Vitis vinifera*, Allergen, Bronchial asthma, Lung function, Histamine

## Abstract

**Background:**

Fruits of *Vitis vinifera* L., commonly known as grapes, are largely consumed worldwide because of their high nutritional and medicinal benefits.

**Context and purpose:**

The present study investigated effects of *V. vinifera* fruits in ovalbumin-induced animal model of bronchial asthma.

**Methods:**

Male wistar rats (except group 1) were sensitized with allergen (ovalbumin, 40 mg/rat + aluminum hydroxide, 2 mg/rat). Groups of sensitized animals were treated orally with either vehicle (0.4 mL/kg), standard dexamethasone (2.5 mg/kg) or alcoholic extract of *V. vinifera* dried fruits (31 and 42.5 mg/kg) from day 1 to 28 (*n* = 6 for all groups). Inflammatory markers including cell counts, cytokines such as interleukin (IL)-4, IL-5, IL-1β, tumor necrosis factor, immunoglobulin E (IgE), leukotrienes and nitrite levels in both blood/serum and bronchoalveolar fluid were analysed. Breathing rate and tidal volume as lung function parameters were examined by spirometer. Lung tissues were studied for histamine content and histopathology.

**Results:**

Treatment of sensitized animals with dexamethasone or two doses of *V. vinifera* fruits extract inhibited recruitment of inflammatory cytokines, IgE, nitrites and circulating cells particularly eosinophils in blood/serum and bronchoalveolar fluid (*p* < 0.001, *p* < 0.01 and *p* < 0.05). Dexamethasone and *V. vinifera* fruits extract treatment also normalized lung functions and histamine levels compared to ovalbumin-sensitized controls (*p* < 0.05 and *p* < 0.01). Moreover, both drugs exhibited protection against airway inflammation in lung histology.

**Conclusion:**

Results of study demonstrate the effectiveness of *V. vinifera* fruits in allergic asthma possibly related to its ability to inhibit cellular response and subsequent production of inflammatory cytokines.

**Electronic supplementary material:**

The online version of this article (doi:10.1186/s13223-016-0145-x) contains supplementary material, which is available to authorized users.

## Background

*Vitis vinifera* (Family: Vitaceae), commonly known as grapes, are widely utilized as natural dietary supplements due to their unique phytochemical composition and high nutritional value. Fruits are a good source of polyphenols [1], anthocyanins [2], flavanols [3], stilbenes (resveratrol) [4], phenolic acids, proteins, fats, vitamins (C and A) [5], minerals (calcium, boron phosphorus) [6], water, carbohydrates and fibers [7]. The medicinal value of the plant has long been recognized in folklore medicine. Documented evidences report anti-diabetic, cytotoxic [8], anti-aging [9], cardioprotective [10], hypolipdemic [11], anti-inflammatory [12] and antioxidant [13] properties of seeds and fruits of *V. vinifera*.

Regarding inflammatory process in chronic airway diseases such as asthma and chronic obstructive pulmonary disorder, disease pathology is directly associated with increased generation of reactive oxygen species in the lungs [14]. Several clinical studies suggest that supplementation of antioxidants benefit adults with mild to moderate asthma [15, 16]. Since, oxidative damage plays a significant role in the pathology of bronchial asthma therefore this process may represent a potential target of the management therapy in asthmatic patients [17].

Asthma is a chronic inflammatory disease of airways characterized by reversible constriction of the tracheobronchial tree and airway hyperresponsiveness to various stimuli such as environmental allergens, respiratory infection, cold air, exercise and some drugs [18, 19]. In human beings, allergic asthma is primarily initiated by a type I hypersensitivity reaction which represents increased susceptibility to produce immunoglobulin E (IgE) in response to external allergens, secretion and differentiation of which is dependent on CD4+ helper T cells (T_H_2-type) [20, 21]. Activated T_H_2 cells produce a number of cytokines such as interleukin (IL)-3, IL-4, IL-5, IL-13 which in turn play role in various process such as promoting production of IgE cells by B cells, growth of mast cells (IL-4) and survival of eosinophils by IL-5 [22]. IL-4 and IL-13 also stimulate epithelial cells to produce transforming growth factor alpha (TGF-α) which causes mucosal metaplasia and fibroblast proliferation [23]. Pro-inflammatory mediators including tumor necrosis factor (TNF) and granulocyte macrophage colony-stimulating factor (GM-CSF) stimulate the expression of vascular adhesion molecules on endothelial cells which result in an increased influx of inflammatory leukocytes into the bronchial tree [24].

With regard to presence of rich plethora of phenolic antioxidant constituents such as gallic acid and resveratrol in *V. vinifera*, the present study was conducted to examine therapeutic potential of dried fruits of *V. vinifera* in ovalbumin induced rat model of allergic asthma.

## Methods

### Drugs and chemicals

Ovalbumin (OVA), dexamethasone, gallic acid, heparin, methacholine, vecuronium bromide were purchased from Sigma Aldrich, St. Louis, MO, USA. Standard ELISA kits used for the determination of rat interleukin (IL)-4, tumor necrosis factor (TNF), IL-1β (Ray Biotech, Inc., IL, USA), IgE (Immunology Consultants Laboratory, Inc., Portland, OR), IL-5 and leukotrienes LTD_4_ (Cusabio Biotech, Hubei, China), Nitric oxide (NO) calorimetric kit (BioVision Research Products, USA) were purchased from commercial suppliers. All other chemicals were commercial products of analytical reagent grade.

### Collection of plant and preparation of extract

*Vitis vinifera* L. dried fruits were collected from a local Indian supplier and botanically authenticated by Dr. H. B. Singh at National Institute of Science Communication and Information Resources, India. A sample voucher NISCAIR/RHMD/consult/-2011-12/1752/52) was submitted in herbarium of Jamia Hamdard, Hamdard University, New Delhi, India, for future reference. Dried fruits (1000 g) were homogenized and exhaustively extracted with ethanol for 3 days at 32 ± 2 °C. The extract was separated by filtration and concentrated in rotary vacuum evaporator (Buchi, USA) and then dried in lyophilizer (Uni-step, India, model: PPI –SX72) under reduced pressure. The yield obtained was 426.44 g of viscous dark brown residue (yield 42.64 % w/w). 250 mg extract was dissolved in purified water with the help of carboxymethyl cellulose (0.1 %). The prepared suspension of alcoholic extract of *V. vinifera* (VVHE) dried fruits was stored in refrigerator till administration to animals.

### Quantification of gallic acid by HPTLC

#### Preparation of sample and standards

10 mg of residue was dissolved in 1 mL of ethanol to obtain the concentration of 10 mg/mL. Stock solutions of gallic acid were prepared by dissolving 1 mg of gallic acid in 1 mL of ethanol and making dilutions to get the final concentration, 100 µg/mL of standard. Sample and standard solutions were filtered through a 0.22 µM membrane filter.

#### Chromatographic conditions

For quantifying gallic acid in ethanol extract of *V*. *vinifera* dried fruits, mobile phase with composition toluene: ethyl acetate: formic acid: methanol (3.5:3.5:0.8:0.5) and chamber saturation time 35 min was used. The standard and sample were spotted in the form of bands (width 8 mm with a CAMAG microliter syringe) on a pre-coated silica gel plate 60F-254 aluminum sheets (20 × 10 cm with 0.2 mm thickness; Merck KGaA, Darmstadt, Germany) using a CAMAG Linomat-V applicator (Muttenz, Switzerland) attached to CAMAG HPTLC system. The plates were pre-washed with methanol and activated at 105 °C for 5 min prior to chromatography. The standard and sample-loaded plates were kept in the TLC twin trough developing chamber (after saturating with solvent vapor for 15 min at 28 ± 2 °C) with respective mobile phase till plate run up to 80 mm. The developed plates were dried in hot air to evaporate solvents and scanned at 292 nm with CAMAG TLC densitometric scanner 3 operated by WinCATS software, using deuterium lamp. To quantify gallic acid 10 µl/spot of sample solution was applied on HPTLC plates.

#### Calibration curves for standards

From the stock solution of standard gallic acid (100 μg/mL) different volumes 1, 2, 4, 6, 8 and 10 μl, were spotted on a precoated TLC plate to obtain corresponding concentrations of 100, 200, 400, 600 and 800 and 1000 ng/spot of standard. Each application was done in triplicate. The regression equation for gallic acid was y = (1216 ± 2.863) + (6.484 ± 0.007)x; (Co-relation coefficient, r^2^ = 0.996 ± 0.0084).

#### Animals

The guidelines of Government of India regarding control and supervision of experiments on animals were followed. Approval was obtained from the Institutional Animal Ethics Committee (IAEC) before conducting the present study (Registration no. 173/CPCSEA/748). Male wistar rats (180–220 g; 8–10 weeks old) were procured from Central Animal House, Jamia Hamdard, New Delhi. Animals were housed in polypropylene cages under controlled conditions (room temperature 25 ± 2 °C; humidity 45 ± 5 %; and photoperiod of 12 h light: dark cycle). All the animals had free access to standard laboratory conditions of diet and water ad libitum throughout the study.

#### Experimental design

After acclimatization of male wistar rats to the standard housing conditions, animals were randomly divided into 5 groups (6 rats per group). Group 1 (SAL), the non-sensitized control, received vehicle (0.4 mL/kg); Group 2 (OVA), the OVA-sensitized or asthma control, was OVA-sensitized receiving vehicle only; Group 3 (OVA + dex), reference standard was OVA-sensitized received dexamethasone (2.5 mg/kg, b.w.); Group 4 and 5, the experimental groups (OVA + VVHE 1 and OVA + VVHE 2) were OVA-sensitized and received alcoholic extract of VVHE (31 and 42.5 mg/kg, b.w., respectively). *Vitis vinifera* fruits are an important constituent of various herbal formulations mentioned in Ayurvedic Pharmacopoeia of India (API). In view of this, dose of extract was calculated from human dose of drug mentioned in API [25]. Drugs or vehicle were administered orally from day 1 to 28 once daily in the morning hours.

Asthma was induced in rats using the method of Abdureyim et al., with some modifications (2011) [26]. All animals (except Group 1) were sensitized with intraperitoneal injection of allergen suspension (ovalbumin, 40 mg/rat + aluminum hydroxide, 2 mg/rat) on day 1. After 15 days of sensitization, animals were challenged by exposure to aerosol consisting of 1 % ovalbumin in normal saline for 20 min. Animals in the non-sensitized group were exposed to saline following the same protocol. Exposure to aerosolized solutions were done in a closed chamber (dimensions 40 × 32 × 32 cm) once daily for 8 consecutive days i.e., from day 15 to day 22, and thereafter, on day 25 and day 28.

#### Lung function and bronchoconstriction test

On day 28, 5 min after OVA exposure, rats were anaesthetized with an intraperitoneal injection of sodium pentobarbitone (105 mg/kg). Trachea was cannulated using a 12G cannula (2 mm internal diameter) as described previously [27]. Cannula was connected to a pneumotachograph (spirometer, Model no: FE141, ADInstruments, Pty, Australia) with attached flow head (Model: MLT 10L, 10 L/min, suitable for rats, ADInstruments, Pty, Australia) and differential pressure transducer to measure respiratory rate (*f*, breaths/min). Lungs tidal volume (V_T_, mL/s) were obtained by electronic integration of airflow signal. Data was stored in Power Lab System (ADInstruments, Pty, Australia) and acquired through LabChart Programme installed in the lab’s computer. Spirometer was calibrated before use to record data in terms of L/s and any drift or offsets in signal due to transducer was nullified to zero for accurate measurements. Femoral vein was cannulated with a 24G needle cannula filled with heparin for administration of methacholine **(**1.5 mg/kg). Changes in *f* and V_T_ were recorded (1) before and after vehicle administration; (2) before and after methacholine treatment. 10–12 respiratory cycles were averaged to provide one data point. Vecuronium bromide (0.2 mg/kg) was injected intravenously to avoid incidence of spontaneous respiration. Excessive bronchial secretions were discharged using a small polyethylene tube without disturbing the trachea [28].

#### Bronchoalveolar lavage (BAL) fluid collection

Immediately after measurement of lung function parameters, lungs were lavaged for 3 times with 5 mL (5 mL × 3) aliquots of 0.9 % sterile saline solution via cannulated tracheal tube [29]. The BAL fluid recovered from each rat was pooled separately (approximately 11–12 mL/rat) and centrifuged (1500 rpm, 10 min at 4 °C). Supernatant was separated and stored at −80 °C until analysis for IgE and cytokines, whereas cell pellet was re-suspended in 1 mL of physiological saline for determination of total and differential leukocyte count as per method described by Jung et al. [30].

#### Serum preparation and cell count

Following collection of BAL fluid, blood was collected by cardiac puncture in two different portions. The first aliquot (2.5–3 mL) was collected in a non-heparinized tube; centrifuged (3000 rpm, 10 min) and serum stored at −80 °C for estimation of IgE and cytokines. The second aliquot (0.5 mL) was collected in a heparinized tube and stored at 4 °C until determination of total cell and differential count.

Within 30 min of collection of heparinized blood, total leukocyte count and differential cell count in blood was determined by autoanlyser (XP-100, Sysmex Corporation, Japan). Differential cell count was made from cytospin smears stained with Leishman’s stain (1.5 % in methanol for 6 min). A minimum of 500 cells were counted under an optical digital microscope (B1 series system, 400× magnification) and classified into eosinophils, neutrophils, lymphocytes, macrophages or monocytes using standard morphologic criterion. Absolute number of each cell type was calculated.

#### IgE, LTD_4_, cytokines, nitric oxide and nitrite levels in serum and BAL fluid

The levels of IgE, LTD_4_ and cytokines (TNF, IL-4, IL-5 and IL-1β) in serum (500 μl) and BAL fluid (5 mL) were measured using enzyme-linked immunosorbent assay (ELISA) kits. The samples were analyzed on an automated ELISA plate reader (Model no. ELX-80MS, Biotek, USA). Concentration of total NO and nitrite levels in serum and BAL fluid were determined by nitric oxide calorimetric kit.

#### Histamine analysis in lavaged lung tissue

After collection of BAL fluid lung tissue lobes from each animal were separately dissected out. One of the lobes was homogenized with 2.5 mL normal saline and processed for analysis of histamine as per method described by Shore et al., using spectrophotometer at 650 nm [30, 31].

#### Histological examination

Dissected lung tissues were washed with normal saline (NS) and fixed in 10 % neutral formaldehyde solution at 4 °C for 24 h. The specimens were embedded in paraffin wax and sectioned to 5 µm with a rotary microtome. Sections were stained with 1 % hematoxylin in distilled water and 1 % eosin dye in 90 % alcohol (H & E) for studying morphology. Tissues were mounted with glycerin jelly and cover slipped. The slides were examined under Labcon trinocular research microscope and photographs were taken with a Nikon Coolpix digital zoom camera (model no. S3300).

#### Ethics aspects

The research was approved by Institutional Animal Ethics Committee (IAEC) before conducting the present study under Registration no. 173/CPCSEA/748.

### Statistical analysis

Results were reported as mean ± SEM. Statistical analyses were performed using one-way analysis of variance (ANOVA) followed by post hoc Tukey’s test. Differences were considered statistically significant at value of *p* < 0.05. All statistical analyses were performed using the Graph Pad software (San Diego, CA, USA).

## Results

### Quantification of gallic acid by HPTLC in *V. vinifera* dried fruits

The finger print profile of ethanol extract of *V. vinifera* dried fruits in developed solvent system is shown in Additional file 1: Figure S1. The ethanolic extract of *V. vinifera* showed 8 peaks where fifth peak at R_*f*_ values 0.43 ± 0.027 with corresponding area of 2943.73 ± 4.542 coincides with standard gallic acid The mean content of gallic acid was calculated to be 2.66 ± 1.12 mg/gm of VVHE.

The developed analytical method was optimized by applying BBD-RSM (Box-Behnken Design-Response Surface Methodology). Analysis of variance for isolation and quantification of gallic acid confirmed that the analytical model was statistically significant with F-value 32.55 and depicted by the value of Prob > F less than 0.05. Quadratic equation generated from experimental parameters (Additional file 1: Table S1) showed a reasonable agreement of predicted R^2^ (0.8418) with the adjusted R^2^ (0.9404) (Additional file 1: Figures S2, S3) given by the following equations:

$$\begin{aligned} {\text{Y}} & = 2.63 + 0.39{\text{A}} - 0.22{\text{B}} + 0.1{\text{C}} + 0.14{\text{D}} - 0.15{\text{AB}} - 0.22{\text{AC}} - 0.14{\text{AD}} + 0.22{\text{BC}} + 0.27{\text{BD}} \\ & \quad - 0.30{\text{CD}} - 0.51{\text{A}}^{2} - 0.40{\text{B}}^{2} - 0.41{\text{C}}^{2} - 0.33{\text{D}}^{2} \\ \end{aligned}$$

where, A, is amount of toluene (mL); B, is amount of ethyl acetate (mL); C, amount of formic acid (mL); and D, is time for saturation of developing chamber (min).

### Effect of VVHE on lung function parameters

**Fig. 1:**
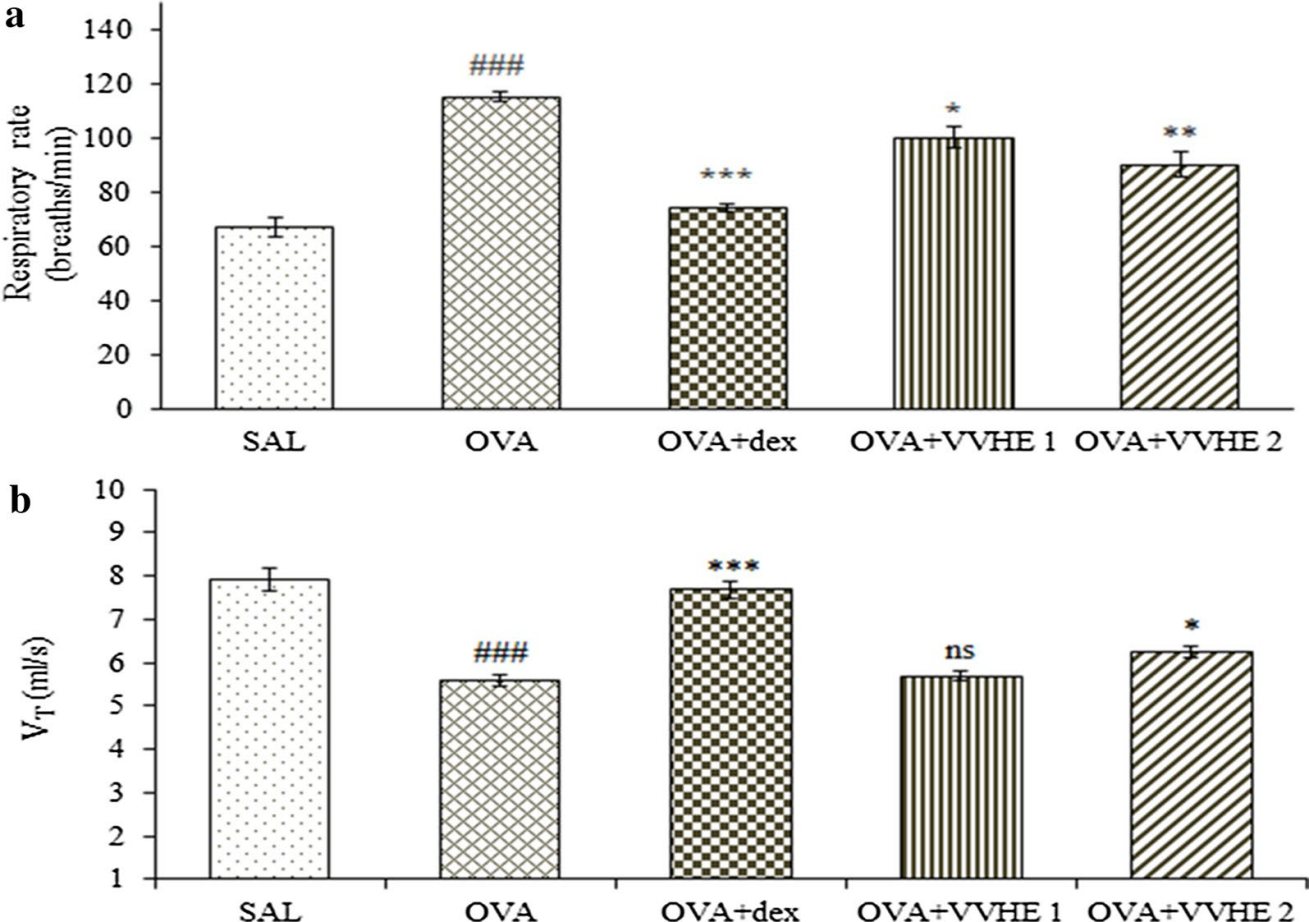
Effect of VVHE on breathing rate (**a**) and tidal volume (**b**) in OVA-sensitized rats. Values shown are mean ± SEM. (*n* = *6*); ^#^ *p* < 0.05, ^###^ *p* < 0.001 and ns (non-significant) compared to the non-sensitized control (SAL); **p < 0.01 and ***p < 0.001, compared to the OVA-sensitized control (OVA). *SAL* non-sensitized and vehicle treated control group, *OVA* OVA-sensitized and vehicle treated control group, *OVA* + *dex* OVA-sensitized and dexamethasone, 2.5 mg/kg, b.w, treated group, *OVA* + *VVHE 1* OVA-sensitized and VVHE, 31 mg/kg, treated group, *OVA* + *VVHE 2* OVA-sensitized and VVHE, 42.5 mg/kg, b.w. treated group

After methacholine injection, respiration rate was significantly (*p* < 0.001) increased in OVA-sensitized control group as compared to non-sensitized control group. Animals receiving VVHE 1 (31 mg/kg) or VVHE 2 (42.5 mg/kg), exhibited 13 or 21 % decrease (*p* < 0.05 or *p* < 0.01) in respiratory rate as compared to the OVA-sensitized control group. There was a considerable reduction in tidal volume (*p* < 0.001) in OVA-sensitized control animals as compared to non-sensitized control. Treating asthmatic rats with VVHE 2 (42.5 mg/kg, b.w.) markedly increased (*p* < 0.05) tidal volume by 11 % as compared to the OVA-sensitized control group ( Fig. 1a, b). Similar comparisons for dexamethasone treated animals exhibited improvement (*p* < 0.001) in breathing rate and tidal volume by 37 and 28 %, respectively.

### Effect of VVHE on circulating cell count in blood

The total number of circulating leukocytes, eosinophils, and neutrophils in blood samples of OVA-control rats were markedly increased (*p* < 0.001) compared to non-sensitized control animals. In contrast, allergen sensitized and challenged rats showed lesser number of lymphocytes in blood compared to normal group animals. Elevated number of eosinophils and neutrophils significantly reduced after treatment with VVHE 2 (42.5 mg/kg, b.w., *p* < 0.05) and dexamethasone (*p* < 0.01) compared to asthma control group. Both drug treatments also normalized the lymphocytes count in blood of animals (Table 1).

**Table 1:** Effect of VVHE on total cells and differential cell count in blood (×10^5^ cells/ml) of ovalbumin-sensitized rats

Groups	Total cells	Eosinophils	Lymphocytes	Macrophages	Neutrophils
SAL	9.52 ± 0.46	0.63 ± 0.06	10.85 ± 0.43	6.40 ± 0.65	0.35 ± 0.08
OVA	17.33 ± 1.14^###^	4.87 ± 0.35^###^	7.15 ± 0.77^##^	6.36 ± 0.68^ns^	4.19 ± 0.46^###^
OVA + dex	11.50 ± 0.43***	1.93 ± 0.27**	10.60 ± 0.36*	6.35 ± 0.31^ns^	1.94 ± 0.26**
OVA + VVHE 1	16.37 ± 0.81^ns^	4.01 ± 0.47^ns^	9.93 ± 1.77^ns^	6.03 ± 0.37^ns^	1.35 ± 0.36^ns^
OVA + VVHE 2	13.83 ± 0.76**	2.91 ± 0.42*	10.30 ± 0.43*	6.10 ± 0.40^ns^	2.42 ± 0.38*

Values shown are mean ± S.E.M. (*n* = *6*)

ns (non-significant) compared to OVA-sensitized control (OVA)

*SAL* non-sensitized and vehicle treated control group, *OVA* OVA-sensitized and vehicle treated control group, *OVA* + *dex* OVA-sensitized and dexamethasone, 2.5 mg/kg, b.w, treated group, *OVA* + *VVHE 1* OVA-sensitized and VVHE, 31 mg/kg, treated group, *OVA* + *VVHE 2* OVA-sensitized and VVHE, 42.5 mg/kg, b.w. treated group

^##^ *p* < 0.01; ^###^ *p* < 0.001 and NS (non-significant) compared to non-sensitized control (SAL)

* *p* < 0.05; ** *p* < 0.01 and *** *p* < 0.001

### Effect of VVHE on inflammatory cellular counts in BAL fluid

The OVA-sensitized control group showed marked increase (*p* < 0.001) in total cells and differential cellular count in BAL fluid samples compared to non-sensitized control group. Number of eosinophils (*p* < 0.01), lymphocytes (*p* < 0.01), macrophages (*p* < 0.01) and neutrophils (*p* < 0.05) reduced significantly in VVHE 2 (42.5 mg/kg, b.w.) and dexamethasone (*p* < 0.001) treated animals compared to OVA-sensitized controls. VVHE at lower dose level (31 mg/kg) also reduced these inflammatory cells in lavaged fluid but the reduction was statistically significant only for lymphocytes and macrophages (*p* < 0.05) (Table 2).

**Table 2:** Effect of VVHE on total cells and differential cell count in bronchial fluid (×10^5^ cells/ml) of ovalbumin-sensitized rats

Groups	Total cells	Eosinophils	Lymphocytes	Macrophages	Neutrophils
SAL	7.00 ± 0.52	0.51 ± 0.56	9.92 ± 0.44	5.13 ± 0.52	0.55 ± 0.05
OVA	11.17 ± 0.87^###^	8.50 ± 0.44^###^	18.37 ± 0.77^###^	12.08 ± 0.77^###^	5.60 ± 0.46^###^
OVA + dex	4.83 ± 0.48***	3.07 ± 0.42***	10.15 ± 0.71***	6.55 ± 0.81***	1.16 ± 0.08***
OVA + VVHE 1	8.11 ± 0.45*	7.11 ± 0.31^ns^	14.15 ± 0.75*	10.02 ± 0.63*	3.27 ± 1.53^ns^
OVA + VVHE 2	5.83 ± 0.48**	3.85 ± 0.43**	12.32 ± 0.28**	7.76 ± 0.19**	4.01 ± 0.39*

Values shown are mean ± SEM (*n* = *6*)

ns (non-significant) compared to the OVA-sensitized control (OVA)

*SAL* non-sensitized and vehicle treated control group, *OVA* OVA-sensitized and vehicle treated control group, *OVA* + *dex* OVA-sensitized and dexamethasone, 2.5 mg/kg, b.w, treated group, *OVA* + *VVHE 1* OVA-sensitized and VVHE, 31 mg/kg, treated group, *OVA* + *VVHE 2* OVA-sensitized and VVHE, 42.5 mg/kg, b.w. treated group

^###^ *p* < 0.001 compared to non-sensitized control (SAL)

* *p* < 0.05, ** *p* < 0.01, and *** *p* < 0.001

### Effect of VVHE on levels of LTD_4_ and cytokines in serum

**Fig. 2:**
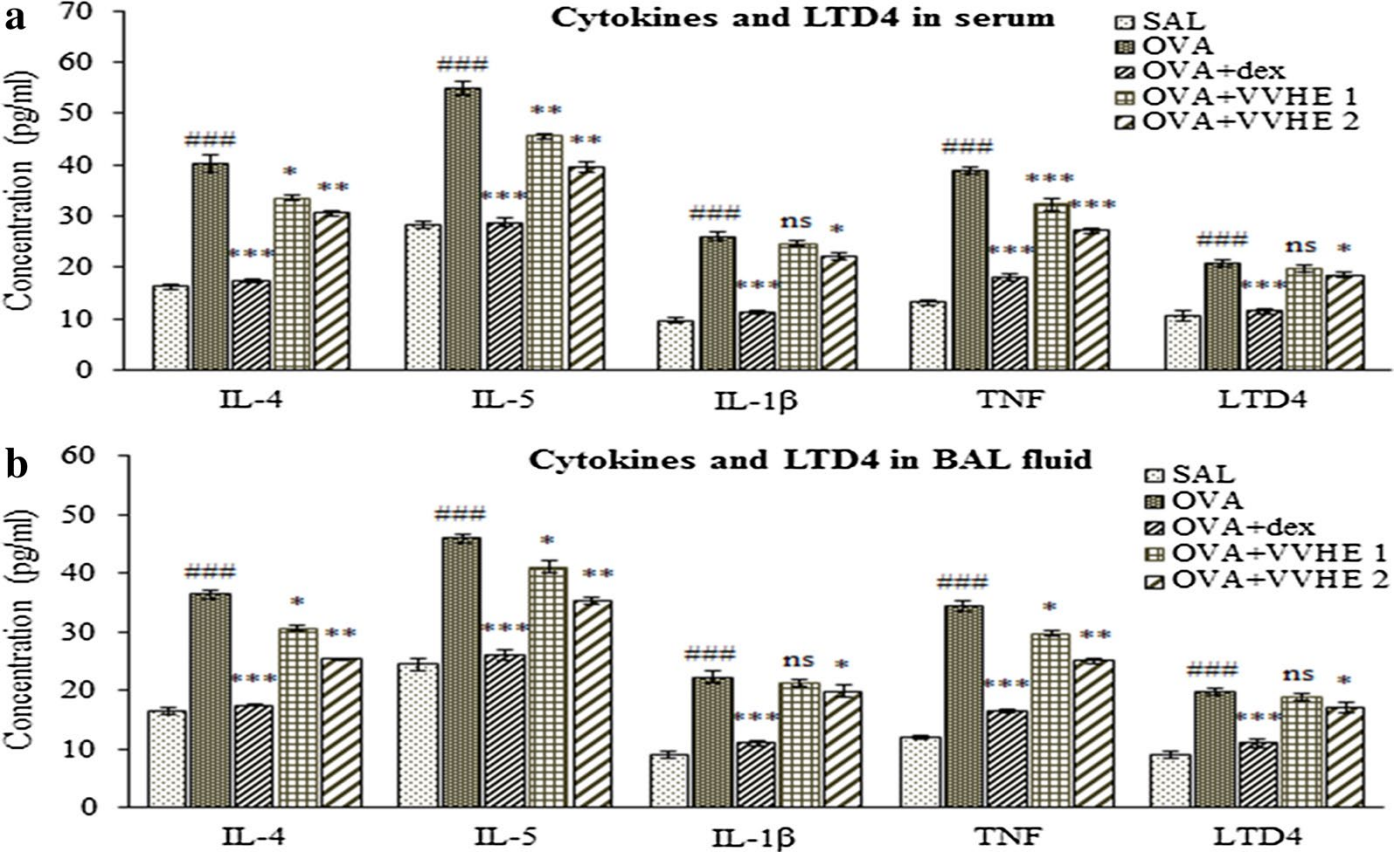
Effect of VVHE on cytokine levels in serum (**a**) and BAL fluid (**b**) of OVA-sensitized rats. Values shown are mean ± S.E.M. (*n* = *6*); ^###^ *p* < 0.001 compared to the non-sensitized control (SAL); *p < 0.05, **p < 0.01, ***p < 0.001, and ns (non-significant) compared to the OVA-sensitized control (OVA). *SAL* non-sensitized and vehicle treated control group, *OVA* OVA-sensitized and vehicle treated control group, *OVA, OVA* + *dex* OVA-sensitized and dexamethasone, 2.5 mg/kg, b.w, treated group, *OVA* + *VVHE 1* OVA-sensitized and VVHE, 31 mg/kg, treated group, *OVA* + *VVHE 2* OVA-sensitized and VVHE, 42.5 mg/kg, b.w. treated group

Significant (*p* < 0.001) elevation in serum levels of LTD_4_ and all the cytokines, IL-4, IL-5, TNF and IL-1β was observed in OVA-sensitized control rats compared to the non-sensitized group controls. VVHE 1 (31 mg/kg, b.w.) and VVHE 2 (42.5 mg/kg, b.w.) treatment reduced serum levels of IL-4 by 17 and 24.2 % (*p* < 0.01), IL-5 by 17.1 and 28.2 % (*p* < 0.01), TNF by 17 and 30.2 % (*p* < 0.001), IL-1β by 5 and 15.3 % (*p* > 0.05 or *p* < 0.05) and LTD_4_ by 4.3 and 11 % (*p* > 0.05 or *p* < 0.05) compared to the OVA-sensitized control group (Fig. 2a). However, treating OVA-sensitized animals with dexamethasone showed significant decrease (*p* < 0.001) in serum levels of LTD_4_ by 44.7 % and all the cytokines tested in the study, IL-4 by 56.6 %, IL-5 by 47.6 %, TNF by 53.3 % and IL-1β by 57.11 %.

### Effect of VVHE on levels of LTD_4_ and cytokines in BAL fluid

The levels of all the cytokines tested in BAL fluid of OVA-control group were significantly high (*p* < 0.001) compared to non-sensitized group animals. Substantial reduction in levels of cytokines by 30.5 % for IL-4 (*p* < 0.01); 23.2 % for IL-5 (*p* < 0.01); 10.6 % for IL-1β (*p* < 0.05); 27.3 % for TNF (*p* < 0.01) and 12.6 % for LTD_4_ (*p* < 0.05) was observed in VVHE 2 (42.5 mg/kg, b.w.) and dexamethasone (43.5-52.1 %, *p* < 0.001) treated groups. Treatment with VVHE 1, lower dose of the extract (31 mg/kg, b.w.) also attenuated all the cytokines and LTD_4_ levels in BAL fluid but inhibition was statistically significant only for IL-4, IL-5 and TNF (*p* < 0.05) (Fig. 2b). In contrast, animals treated with dexamethasone showed significant (*p* < 0.001) reduction in BAL fluid levels of LTD_4_ by 44.2 % and all the cytokines including IL-4 by 52.1 %, IL-5 by 43.5 %, TNF by 52.04 % and IL-1β by 50.5 % compared to OVA-sensitized control group.

### Effect of VVHE on IgE levels of serum and BAL fluid

**Fig. 3:**
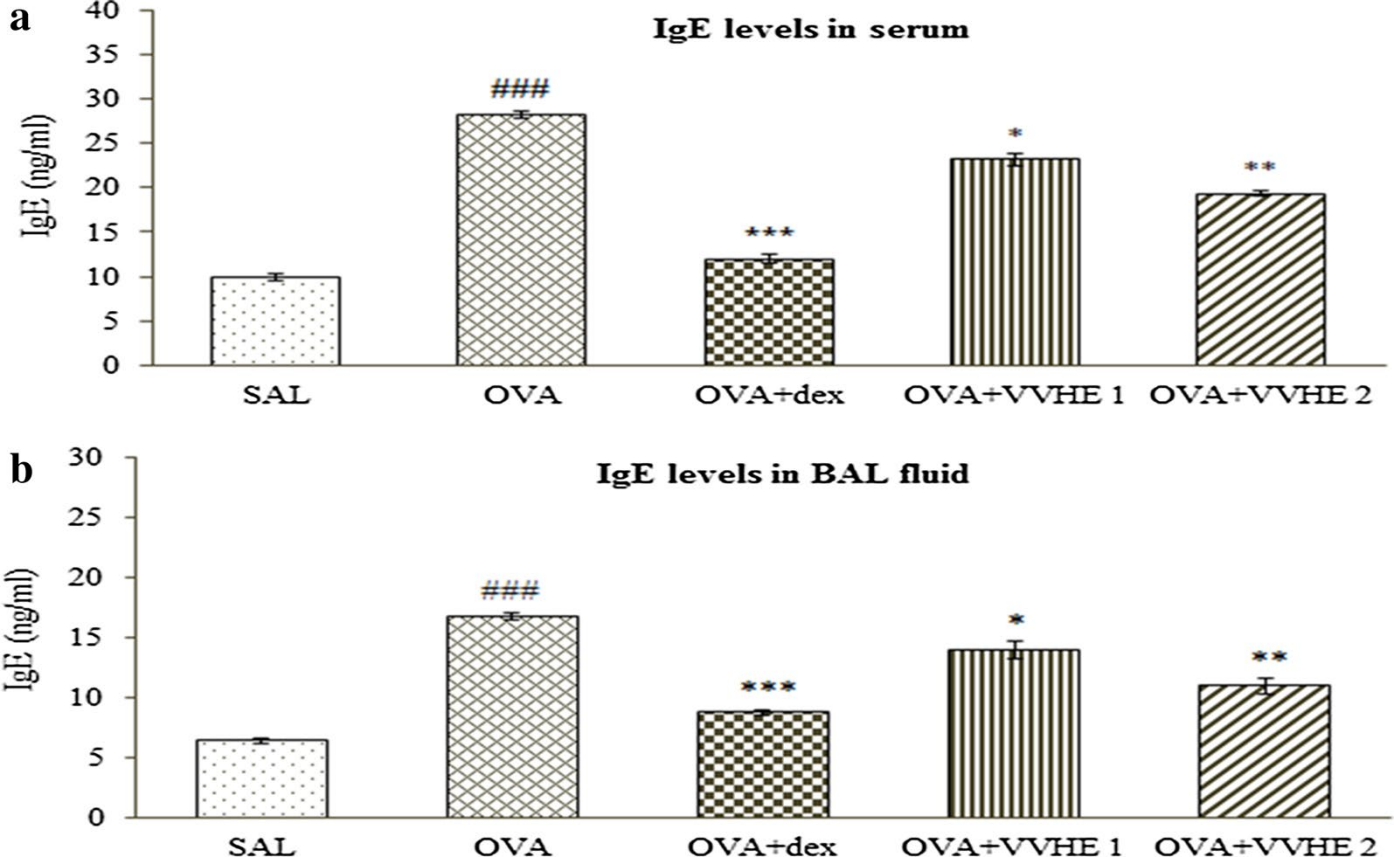
Effect of VVHE on IgE levels in serum (**a**) and BALF (**b**) of OVA-sensitized rats. Values shown are mean ± SEM. (*n* = *6*); ^###^ *p* < 0.001 compared to the non-sensitized control (SAL); *p < 0.05, **p < 0.01 and ***p < 0.001, compared to the OVA-sensitized control (OVA). *SAL* non-sensitized and vehicle treated control group, *OVA* OVA-sensitized and vehicle treated control group, *OVA, OVA* + *dex* OVA-sensitized and dexamethasone, 2.5 mg/kg, b.w, treated group, *OVA* + *VVHE 1* OVA-sensitized and VVHE, 31 mg/kg, treated group, *OVA* + *VVHE 2* OVA-sensitized and VVHE, 42.5 mg/kg, b.w. treated group

Allergen sensitization and challenge resulted in significant rise (*p* < 0.001) in IgE levels of both serum and BAL fluid samples of rats compared with non-sensitized control group. Compared to OVA-control group, animals receiving VVHE 1, 31 mg/kg, b.w., or VVHE 2, 42.5 mg/kg, b.w., exhibited substantial reduction by 17 % or 31 % in serum (*p* < 0.01**)** and 14 % or 26 % in BAL fluid (*p* < 0.01). Treatment with dexamethasone also caused significant (*p* < 0.001) reduction of IgE levels by 58 % in serum and 47.8 % in BAL fluid tested in the study compared to OVA-control group (Fig. 3a, b).

### Effect of VVHE on total nitric oxide and nitrite concentration

**Fig. 4:**
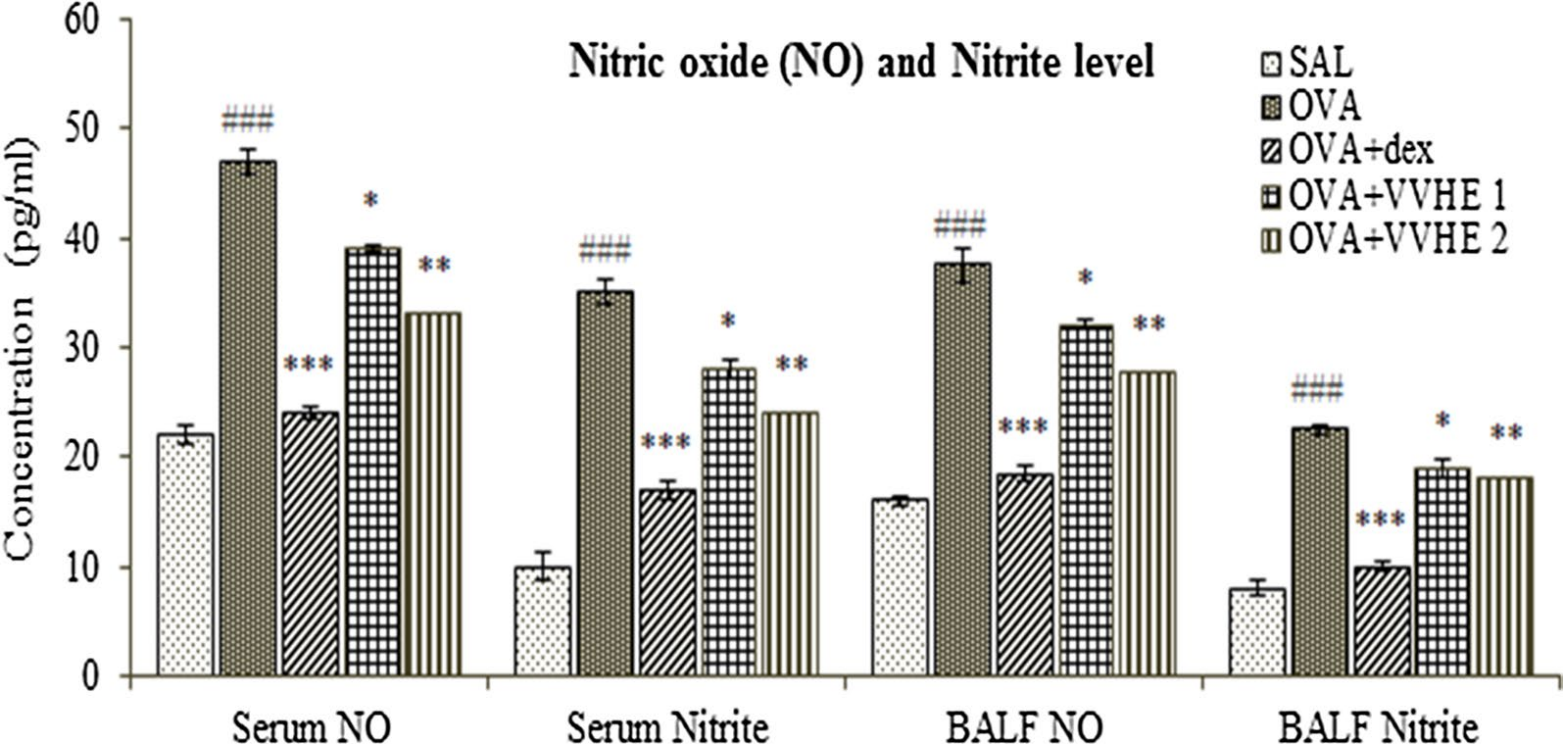
Effect of VVHE on nitric oxide (NO) and nitrite level in serum and BALF of ovalbumin-sensitized rats. Values shown are mean ± S.E.M. (*n* = *6*); ^###^ *p* < 0.001 compared to the non-sensitized control (SAL); *p < 0.05, **p < 0.01 and ***p < 0.001, compared to the OVA-sensitized control (OVA). *SAL* non-sensitized and vehicle treated control group, *OVA* OVA-sensitized and vehicle treated control group, *OVA, OVA* + *dex* OVA-sensitized and dexamethasone, 2.5 mg/kg, b.w, treated group, *OVA* + *VVHE 1* OVA-sensitized and VVHE, 31 mg/kg, treated group, *OVA* + *VVHE 2* OVA-sensitized and VVHE, 42.5 mg/kg, b.w. treated group

A marked (*p* < 0.001) elevation of total NO and nitrite levels were observed in OVA-sensitized control animals as compared to non-sensitized control group. Compared with the asthmatic group animals receiving VVHE 2 (42.5 mg/kg, b.w.) showed 29.7 % and 31.4 % reduced levels of both nitric oxide and nitrites, respectively, in serum and 15 % and 27 % in BAL fluid samples (*p* < 0.01) (Fig. 4). Lower dose of extract (VVHE 1) also decreased (*p* < 0.05) levels of these analytes in both the body fluids. Treatment with reference drug, dexamethasone reduced (*p* < 0.001) elevated serum levels of total NO by 48.8 % and nitrites by 51.5 % and BAL fluids levels by 51 % and 55.6 %, respectively, compared to OVA-sensitized control animals.

### Effect of VVHE on histamine levels in lung tissues

**Fig. 5:**
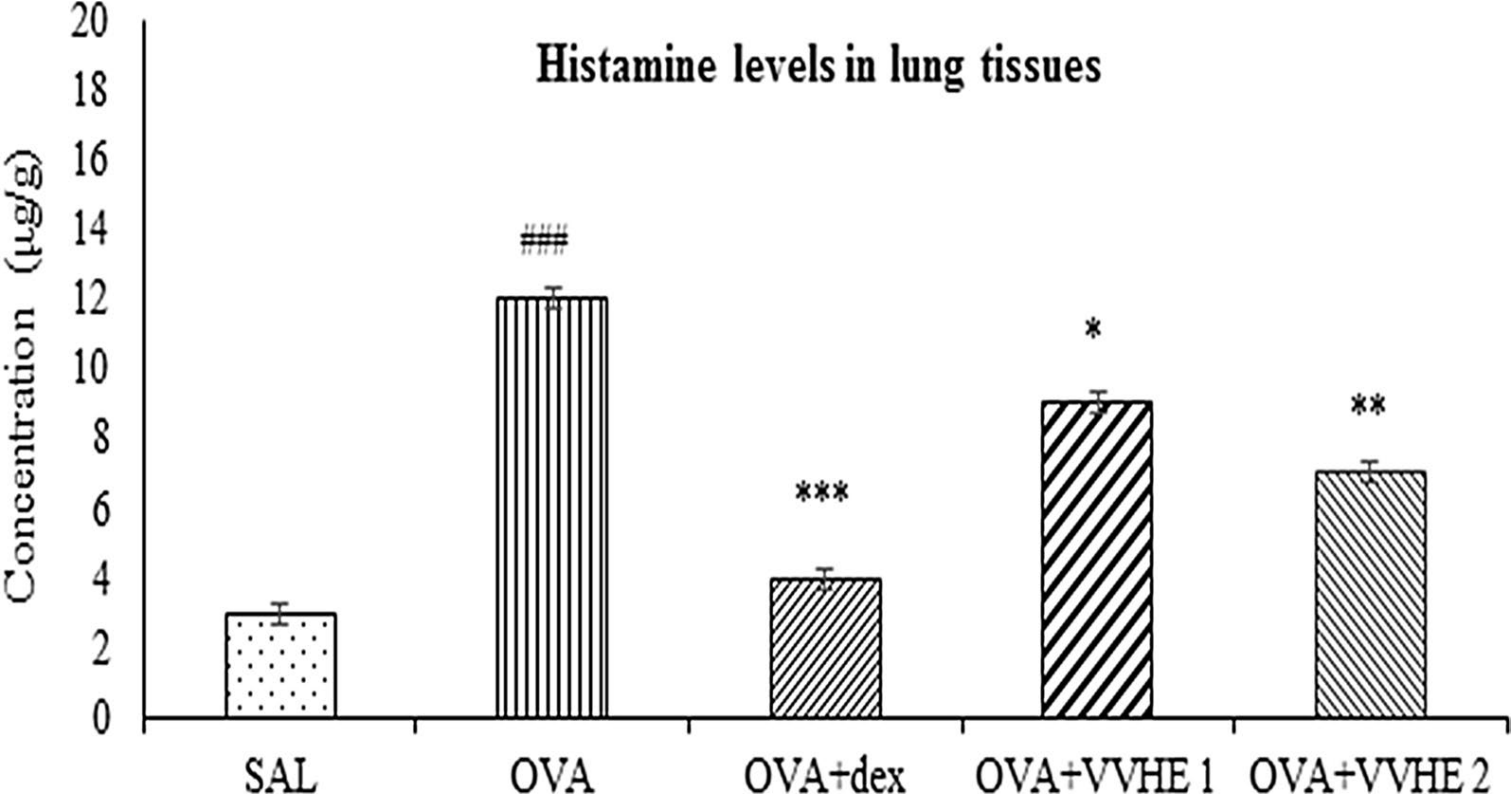
Effect of treatments on lung tissue histamine levels in ovalbumin-sensitized rats. Values shown are mean ± SEM. (*n* = *6*); ^###^ *p* < 0.001 compared to the non-sensitized control (SAL); *p < 0.05, **p < 0.01 and ***p < 0.001, compared to the OVA-sensitized control (OVA). *SAL* non-sensitized and vehicle treated control group. *OVA* OVA-sensitized and vehicle treated control group, *OVA, OVA* + *dex* OVA-sensitized and dexamethasone, 2.5 mg/kg, b.w, treated group, *OVA* + *VVHE 1* OVA-sensitized and VVHE, 31 mg/kg, treated group, *OVA* + *VVHE 2* OVA-sensitized and VVHE, 42.5 mg/kg, b.w. treated group

Analysis of histamine levels in lung tissues homogenates of OVA-control rats were significantly high (*p* < 0.001) compared to non-sensitized controls. VVHE at both dose levels, 31 and 42.5 mg/kg, reduced elevated histamine levels by 25 and 41.6 % (*p* < 0.01) compared with the OVA-control group (Fig. 5). Whereas treatment with standard dexamethasone caused reduction in histamine levels of lung tissue homogenates by 66.6 % compared to that of OVA-control group (*p* < 0.001).

### Effect of treatments on histopathology of lung tissues

**Fig. 6:**
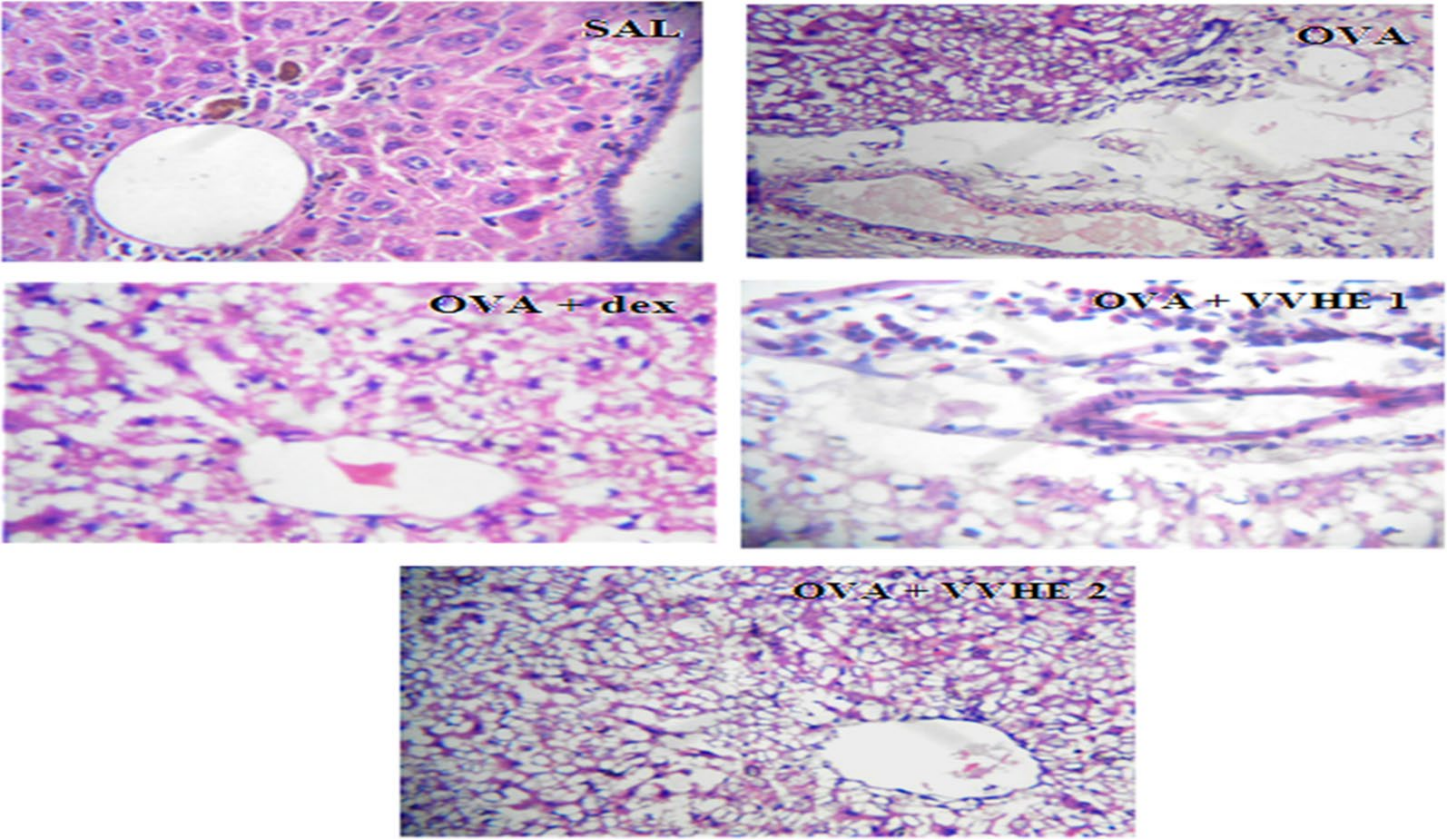
Effect of treatments on the histopathological changes in rat lung tissues. Representative hematoxylin and eosin-stained sections of the rat lungs (×10). *SAL* non-sensitized and vehicle treated control rat showing normal lung histology, *OVA* OVA-sensitized and vehicle treated control rat showing altered bronchiolar lumen and cellular infiltration, *OVA, OVA* + *dex* OVA-sensitized and dexamethasone, 2.5 mg/kg, b.w, treated rat, *OVA* + *VVHE 1* OVA-sensitized and VVHE, 31 mg/kg, treated rat, *OVA* + *VVHE 2* OVA-sensitized and VVHE, 42.5 mg/kg, b.w. treated rat

The histological examination of lung tissues from the OVA-control group showed reduced bronchiolar lumen, infiltration of inflammatory cells into the peribronchial tissues and epithelial desquamation. Treatment with VVHE and standard reference drug, dexamethasone elicited protection against all these pathological features as evidenced by the improvement in lumen size and reduced cellular infiltration (Fig. 6).

## Discussion

Despite all the recent developments, use of herbal drugs is increasing as an adjunctive therapy to the conventional standard treatments in several chronic diseases such as asthma. These health supplements possess a number of medicinal properties which need to be investigated by adapting a translational approach using modern experimental tools. Ovalbumin-induced rat model of asthma is widely used for investigating anti-asthmatic potential of drugs in pre-clinical experimental trials [32, 33]. The main characteristic feature of this model is bronchial hyperresponsiveness to specific stimuli accompanied with airway inflammation and constriction.

In our study, ovalbumin sensitization of rats showed significant increase in breathing rate followed by reduction in tidal volume indicating features of broncho-constriction. Furthermore, all the animals in the asthma control group exhibited sneezing, hyperrhinorrhea and irritability. Protection against methacholine-induced bronchoconstriction in VVHE and dexamethasone treated animals demonstrated the bronchodilatory effect of the drugs. Amelioration of lung functions after treatment with VVHE may be due to reduced airway inflammation resulting in decrease in lung resistance to air flow. Airway hyperresponsiveness with clinical symptoms of high breathing rate and reduced tidal volume is considered as a primary target in the treatment of asthma [34].

Allergen-induced airway hyperresponsiveness is directly associated with T_H_2-driven eosniphilic airway inflammation [35]. Ovalbumin challenge of sensitized rats elicited an increase in number of total cells, eosinophils, and neutrophils in both the body fluids (BAL fluid and serum) compared to that of non-sensitized control group with exception of lymphocyte count in blood. The results were consistent with the study of Schster and group which explained decrease in lymphocytes number may be due to migration of these cells from blood to bronchial fluid after allergen provocation [36]. Oral treatment with VVHE (42.5 mg/kg b.w.) and dexamethasone for 28 days significantly reversed the OVA-induced infiltration of all the inflammatory cells particularly eosinophils, into rat airways, implying the possible role of extract in allergen mediated eosniphilic interventions.

T_H_2 derived inflammatory and pro-inflammatory cytokines such as IL-4, IL-5, TNF and IL-1β are extracellular signaling proteins secreted by almost every cell under certain conditions. These mediators play a critical role in orchestrating all types of inflammatory responses in asthmatic airways [37]. Pharmacological therapies using specific receptors IL-4, IL-5, TNF and IL-1β blockers are likely to constitute a considerable development in asthma management [38, 39]. Present study showed presence of prominent T_H_2 type cytokines in OVA-control group indicating persistent airway inflammation. VVHE at both dose levels 31 and 42.5 mg/kg b.w. for 28 days inhibited elevated levels of IL-4, IL-5, TNF and IL-1β in both serum and BAL fluid compared to OVA-control group. However, changes induced by VVHE were less than that observed in corticosteroid dexamethasone (2.5 mg/kg oral, for 28 days) treated group for inflammation markers in present experiment. Our findings with VVHE suggest that the *V. vinifera* fruits could act in asthma via its neutralizing effects on T_H_2 derived pro-inflammatory (TNF and IL-1β) and inflammatory (IL-4 and IL-5) cytokines, key elements in the pathophysiology of bronchial asthma. Cysteinyl leukotrienes (cys LT’s), LTB_4_, LTC_4_, LTD_4_, and LTE_4_, represent a heterogeneous group of biologically active mediators produced from arachidonic acid. Cys LT’s - D_4_ and E_4_ are stable potent end products which account for clinical features of asthma [40]. VVHE (42.5 mg/kg b.w p.o.) elicited substantial inhibitory effects on cytokine LTD_4_ in both serum and BAL fluid compared to OVA-control animals which may also add to bronchodilation effects of the VVHE in the study.

Increased production of immunoglobulin (IgE) in allergic asthma (atopy) is the strongest detectable predisposing factor in the development of bronchial asthma [41]. The anti-inflammatory effects of VVHE in asthmatic airways were confirmed in our study while evaluating its effect on serum and BAL fluid IgE levels in OVA-sensitized and challenged rats. At both dose levels, VVHE (31 and 42.5 mg/kg, p.o.) was found to be effective in inhibiting the IgE levels in body fluids tested. Marked reduction in levels of both LTD_4_ and IgE was also observed in dexamethasone (2.5 mg/kg oral, for 28 days) treated animals.

NO is a gaseous free radical molecule that is formed by a wide range of cells, including airway and vascular smooth muscle cells, endothelial cells, and epithelial cells nerves, activated macrophages. Inflammatory cells from asthma patients produce more reactive radical species including nitrites than those obtained from normal subjects [42] which perpetuate the ongoing airway inflammation. Some studies report that pH in the asthmatic airways falls during acute condition which facilitates the conversion of nitrite to NO. Hence, increased NO concentrations in the exhaled air of asthmatic patients may reflect nitrite conversion rather than *i*NOS activity [43, 44]. In the present study while evaluating the effects of VVHE on nitric oxide metabolites in serum and BAL fluid of asthmatic rats, VVHE and dexamethasone treatments expressed significant suppression of both NO and nitrite levels in these body fluids after 28 days treatment of OVA-sensitized rats. The data suggests that reduced levels of T_H_2 derived cytokines particularly IL-4 and IL-5 would had inhibited respective secretion of IgE by B cells and infiltration of eosinophils and other inflammatory cells into rat airways leading to suppression of airway inflammation and radical production in lungs.

Polyphenolic compounds present in natural drugs have been reported to elicit potent anti-oxidant and anti-inflammatory properties mediated through inhibition of IL-1β, TNF, COX-2 and production of NO_2_ and PGE_2_ [45–47]. Among them, gallic acid (3, 4, 5-trihydroxy benzoic acid) is one of the most important plant secondary metabolite present in numerous plants including *Vitis vinifera* L. The phytoconstituent is reported to exhibit various biological activities such as antioxidant, anti-inflammatory, anti-diabetic, anti-cancer, anti-microbial, anti-aging, cardio-protective, liver fibrosis [48–52]. In experimental studies on different models of allergic diseases, gallic acid is demonstrated to suppress of allergen induced hypersensitivity reactions in mice and inhibit release of histamine and helper T cell subtypes, IL-4, IL-5 and IL-2 form human mast cells [49, 53, 54]. Accumulating all these data i.e., results of our study on *V. vinifera* fruits and previous findings with gallic acid suggest that amelioration of allergic consequences associated with inflammatory processes in asthmatic rats treated with VVHE, in the present study, may be attributed to the presence of significant amount bioactive constituents including gallic acid in *V. vinifera* fruits.

## Conclusion

From the results of the study, we may suggest that VVHE might play an important role in the management of bronchial asthma by (1) offering protection against ongoing inflammatory process underlying asthma through inhibition of histamine release, cytokine production; (2) improving lung functioning by counteracting allergen induced bronchial hyperresponsiveness; and (3) blocking the release of inflammatory cellular infiltration (eosinophils, lymphocytes, neutrophils) into airways; Further studies may be advised to confirm its use as a valuable anti-asthmatic drug in human beings.

## Additional file

10.1186/s13223-016-0145-x Factors and levels of the Box-Behnken experimental plan. **Figure S1.** HPTLC chromatogram of gallic acid (A) standard and (B) ethanol extract of *Vitis vinifera* L. dried fruits. **Figure S2.** Diagnostic plot representing observed (actual) response values versus the predicted response values in terms of quantification of gallic acid in VVHE. **Figure S3.** Response surface plots representing effects of different independent variables on isolated concentration of gallic acid in VVHE.
