# Supplementary material for: Investigation of anti-asthmatic potential of dried fruits of Vitis vinifera L. in animal model of bronchial asthma
Source: Allergy Asthma Clin Immunol. 2016 Aug 17;12:42. doi: 10.1186/s13223-016-0145-x (PMC4988050; doi:10.1186/s13223-016-0145-x)

**Table S1. Factors and levels of the Box-Behnken experimental plan**

| Factors | Code | Range and levels  Low medium high | | |
| --- | --- | --- | --- | --- |
| Toluene | A | 2.5 | 3.5 | 4.5 |
| Ethyl acetate | B | 2.5 | 3.5 | 4.5 |
| Formic acid | C | 0.4 | 0.8 | 1.2 |
| Saturation time | D | 25 | 35 | 45 |

**Figure S1.**


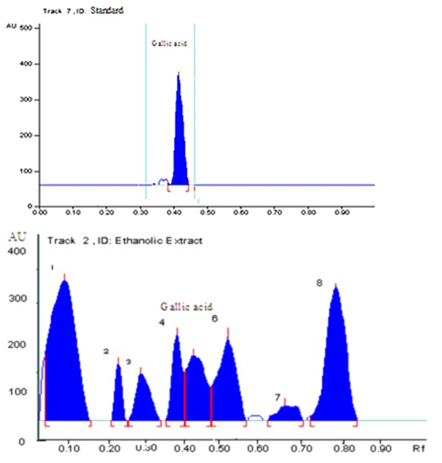


**Figure S2.**

**
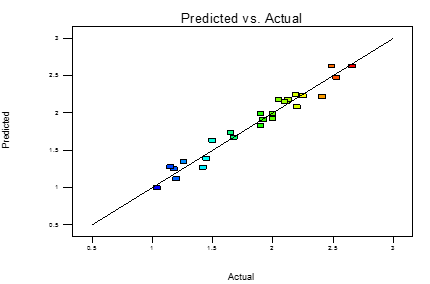
**

**Figure S3.**


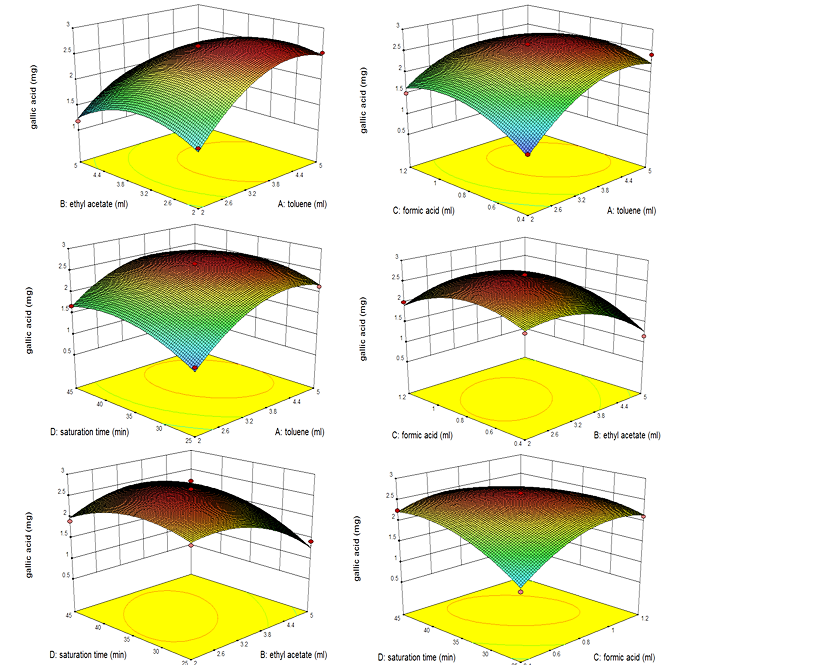

Supplement: Supplementary file 1 — 10.1186/s13223-016-0145-x Factors and levels of the Box-Behnken experimental plan. Figure S1. HPTLC chromatogram of gallic acid (A) standard and (B) ethanol extract of Vitis vinifera L. dried fruits. Figure S2. Diagnostic plot representing observed (actual) response values versus the predicted response values in terms of quantification of gallic acid in VVHE. Figure S3. Response surface plots representing effects of different independent variables on isolated concentration of gallic acid in VVHE. [file 13223_2016_145_MOESM1_ESM.docx]
